# Supplementary material for: Disrupted temperature-sleep coupling mechanism in a Dravet syndrome mouse model
Source: Nat Commun. 2026 Feb 26;17:3232. doi: 10.1038/s41467-026-69957-1 (PMC13062047; doi:10.1038/s41467-026-69957-1)
Supplement: Supplementary file 1 — Supplementary Information [file 41467_2026_69957_MOESM1_ESM.pdf]

Supplementary material

Disrupted temperature-sleep coupling mechanism in a Dravet syndrome mouse model

Saja Fadila, Georgii Krivoshein, Hala Majadly, Anat Mavashov, Shahak Ranen, Marina Brusel, Iria González Dopeso-Reyes, Bertrand Beucher, Eric J. Kremer, Else A. Tolner, Moran Rubinstein

Supplementary Figs. 1 -12

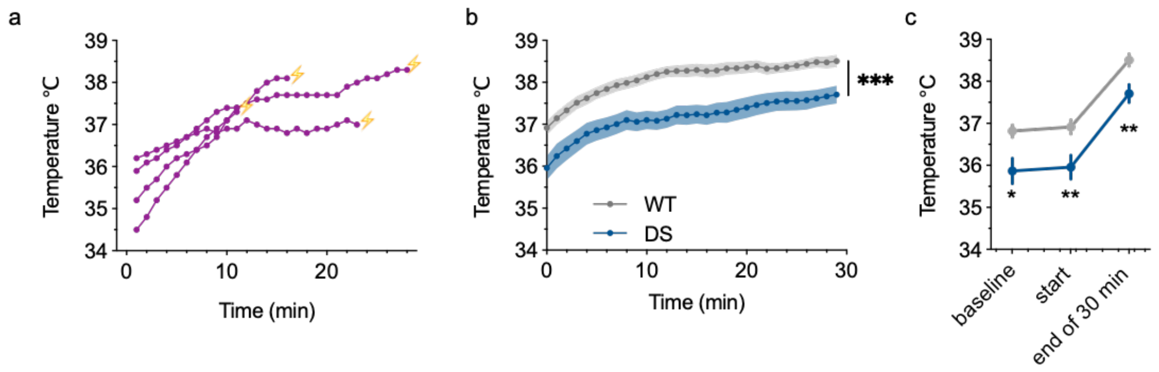

Supplementary Fig. 1

(a). Body core temperature in four DS mice that developed a seizure (marked by lightning) when placed from room temperature to a heated pad warmed to 30°C, yielding an ambient temperature of ~26°C. These are the same mice labeled in magenta in Fig. 1b.

(b). A gradual increase in core temperature was observed over 30 min when the mice were moved from RT to an elevated ambient temperature. The data shown here concern a larger cohort of mice, which includes the 10 WT and 6 DS mice shown in Fig. 1c, as well as 3 additional WT mice (and 6 additional DS mice whose body core temperature was monitored only during the heating phase on a heat pad, but not during the subsequent cooling phase after removal from the pad. (WT: n = 13; DS: n = 12; p = 0.0006, 2-way RM ANOVA).

(c). The change in temperature over the course of the experiment. (p = 0.015, 2-way RM ANOVA, The results of Holm-Sidak post hoc analyses are depicted on the graph). * $p < 0.05$; ** $p < 0.01$; *** $p < 0.001$. Source data are provided as a Source Data file.

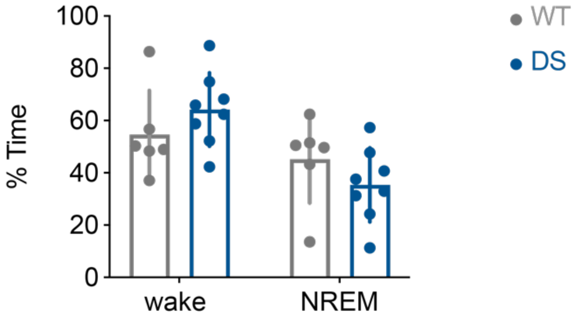

Supplementary Fig. 2
Vigilance state analysis assessed from 2-4 h long ECoG recordings performed at room temperature revealing a similar duration of wakefulness (wake) and NREM sleep in WT and DS mice at the age of 4 weeks. Source data are provided as a Source Data file.

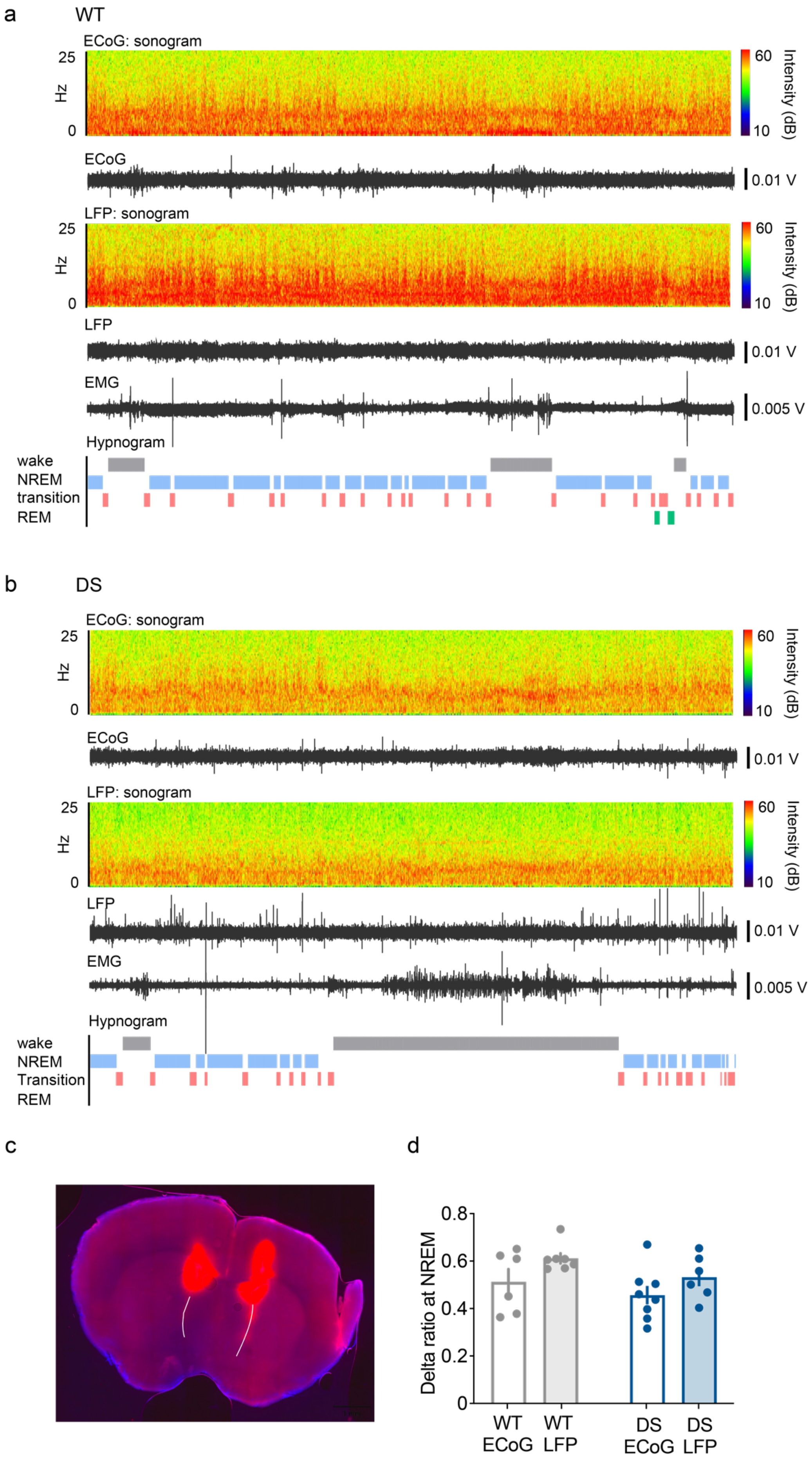
a
WT
ECoG: sonogram
25
Hz
0
60
10
Intensity (dB)
ECoG
0.01 V
LFP: sonogram
25
Hz
0
60
10
Intensity (dB)
LFP
0.01 V
EMG
0.005 V
Hypnogram
wake
NREM
transition
REM
b
DS
ECoG: sonogram
25
Hz
0
60
10
Intensity (dB)
ECoG
0.01 V
LFP: sonogram
25
Hz
0
60
10
Intensity (dB)
LFP
0.01 V
EMG
0.005 V
Hypnogram
wake
NREM
Transition
REM
c
d
Delta ratio at NREM
0.8
0.6
0.4
0.2
0
WT ECoG
WT LFP
DS ECoG
DS LFP

Supplementary Fig. 3
The contribution of the delta frequency band is accentuated in hypothalamic LFP recordings compared to ECoG.
(a) An example of ECoG, hypothalamic LFP, EMG recordings, including sonograms and hypnogram in a WT mouse.
(b) An example of ECoG, hypothalamic LFP, and EMG recordings, including sonograms and hypnogram in a DS mouse.
(c) An example of the LFP electrode positions in the left and right hypothalamic area in a coronal brain section counterstained with DAPI in blue. The electrode wires were coated with DiI (red), and the white lines mark their track.
(d) The contribution of the delta frequency band power to the overall spectral power tended to be higher in LFP recordings compared to ECoG recordings. These data are also depicted in the main Fig. 2d and 2j.
Source data are provided as a Source Data file.

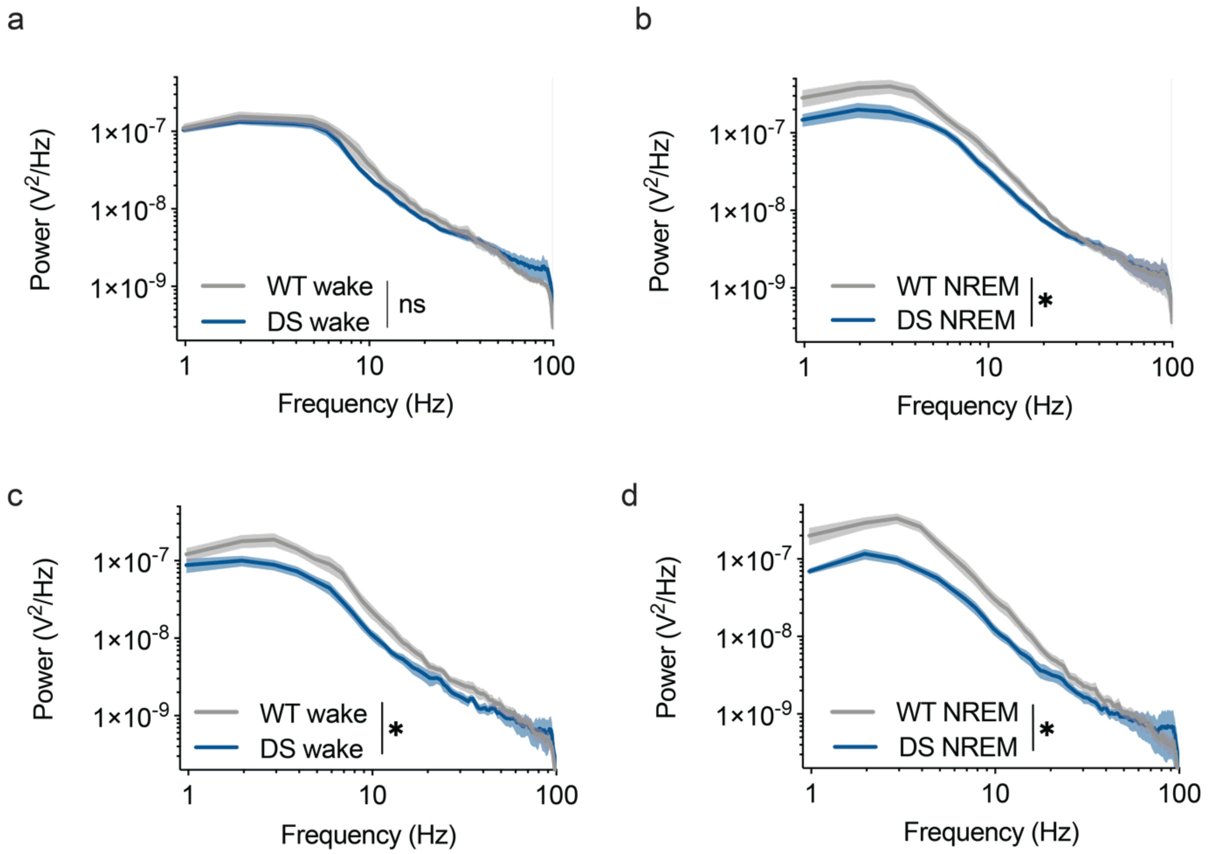

Supplementary Fig. 4

Comparisons of the PSDs of WT and DS mice at wake and NREM, as measured during ~ 4h ECoG or LFP recordings at RT.

(a-b). The same data shown in Fig. 2c, showing PSD from ECoG recordings, are replotted here to highlight genotype differences. (a: $p = 0.3$, b: $p = 0.037$, 2-way ANOVA).

(c-d). The same data shown in Fig. 2i, showing PSD from LFP recordings, are replotted here to highlight genotype differences. (c: $p = 0.04$, d: $p = 0.004$, 2-way ANOVA). Source data are provided as a Source Data file.

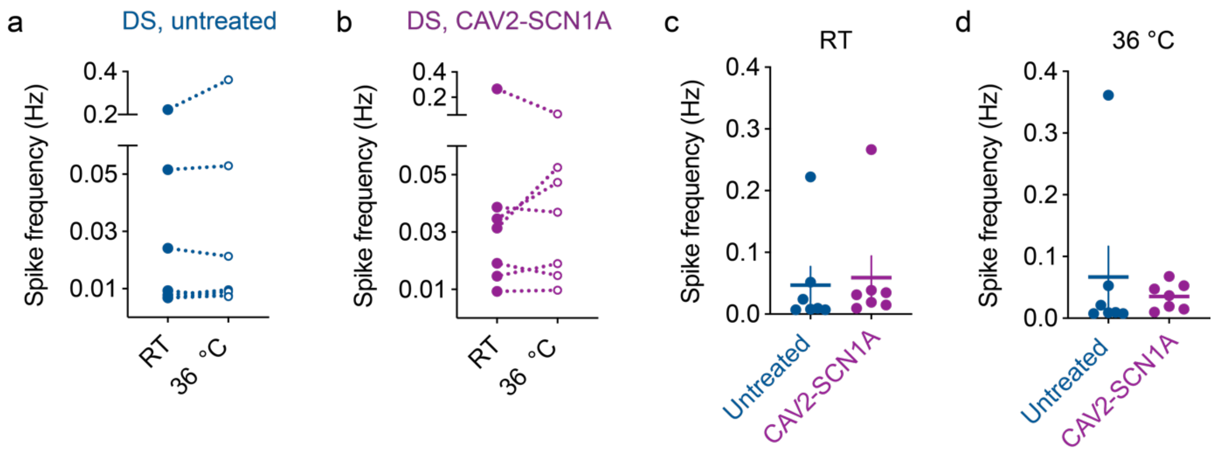

Supplementary Fig. 5

(a, b) For most DS mice, both untreated (n = 7, a) and those treated with the CAV2-SCN1A (n = 7, b), the frequency of interictal spikes did not change when the mice were placed on a heating pad set to 36°C.

(c). A comparison of interictal spike frequency in untreated versus treated DS mice at RT (untreated and treated with CAV2-SCN1A, using the same data depicted in a and b at RT).

(d). A comparison of spike frequency in untreated versus treated DS mice at 36°C (untreated and treated with CAV2-SCN1A, using the same data depicted in a and b at 36°C).

Source data are provided as a Source Data file.

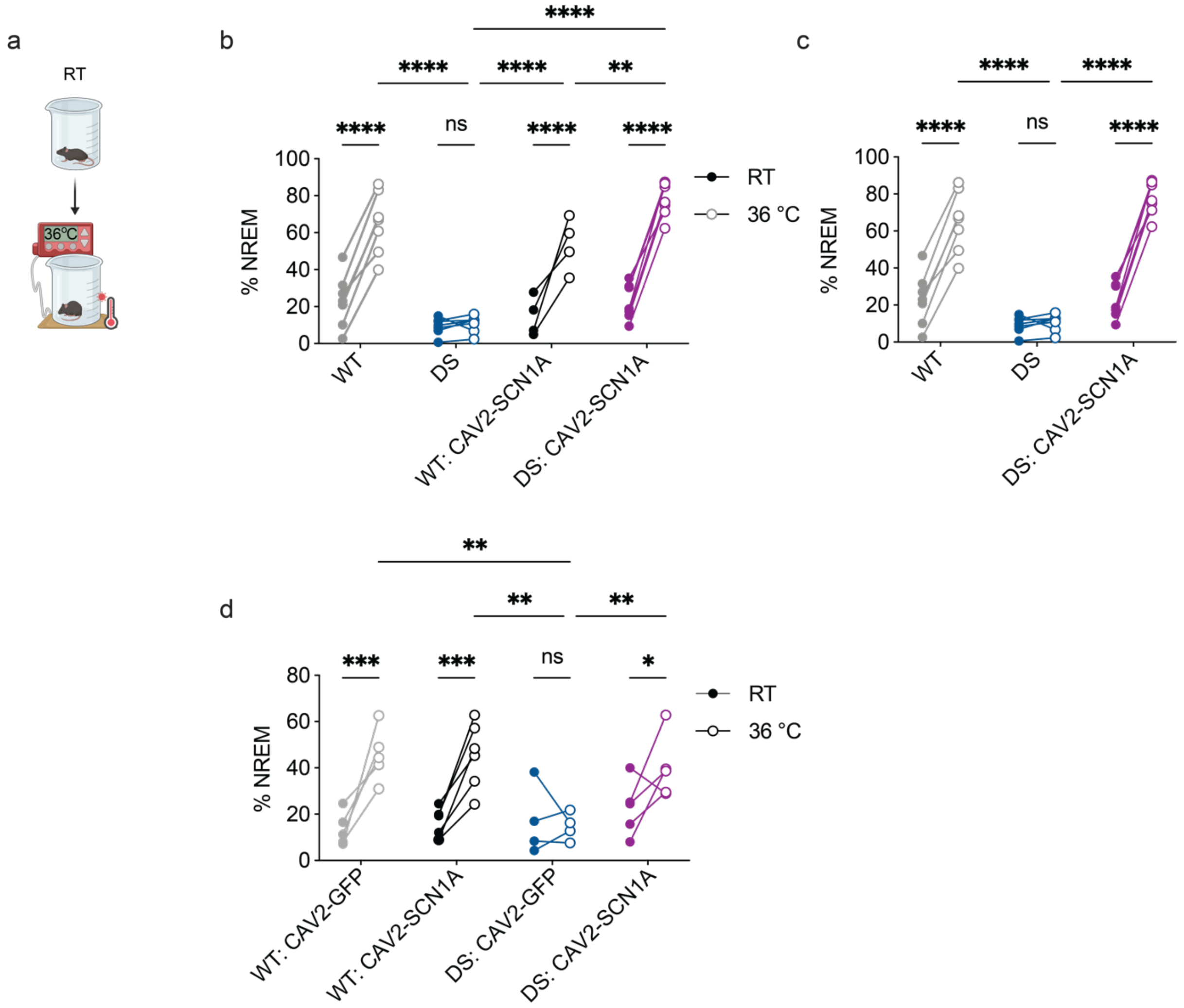

Supplementary Fig. 6
A full statistical comparison between different experimental groups shown in Figs. 3, 4.

(a). The experimental paradigm, used for examining the effect of warmth on sleep promotion.
(b-c). The same data shown in Figs. 3d and 3j are replotted here to highlight genotype differences. (2-way ANOVA. The results of Holm-Sidak post hoc analyses are depicted on the graph).
(d). The data shown in Figs. 4f are shown are depicted again here with the inclusion of the full statistical analysis to highlight also genotype differences. (2-way ANOVA. The results of Holm-Sidak post hoc analyses are depicted on the graph).
* $p < 0.05$; ** $p < 0.01$; *** $p < 0.001$. Source data are provided as a Source Data file.

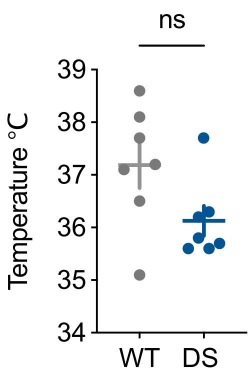

Supplementary Fig. 7
The baseline core temperature of DS mice in their 5th and 6th week of life tended to be lower compared to WT mice but not lower than 35.5°C, unlike the observations in younger DS mice between P21-P25 (see main Fig. 1b) WT: n = 7, DS: n = 7, Mann-Whitney test, $p = 0.08$. Source data are provided as a Source Data file.

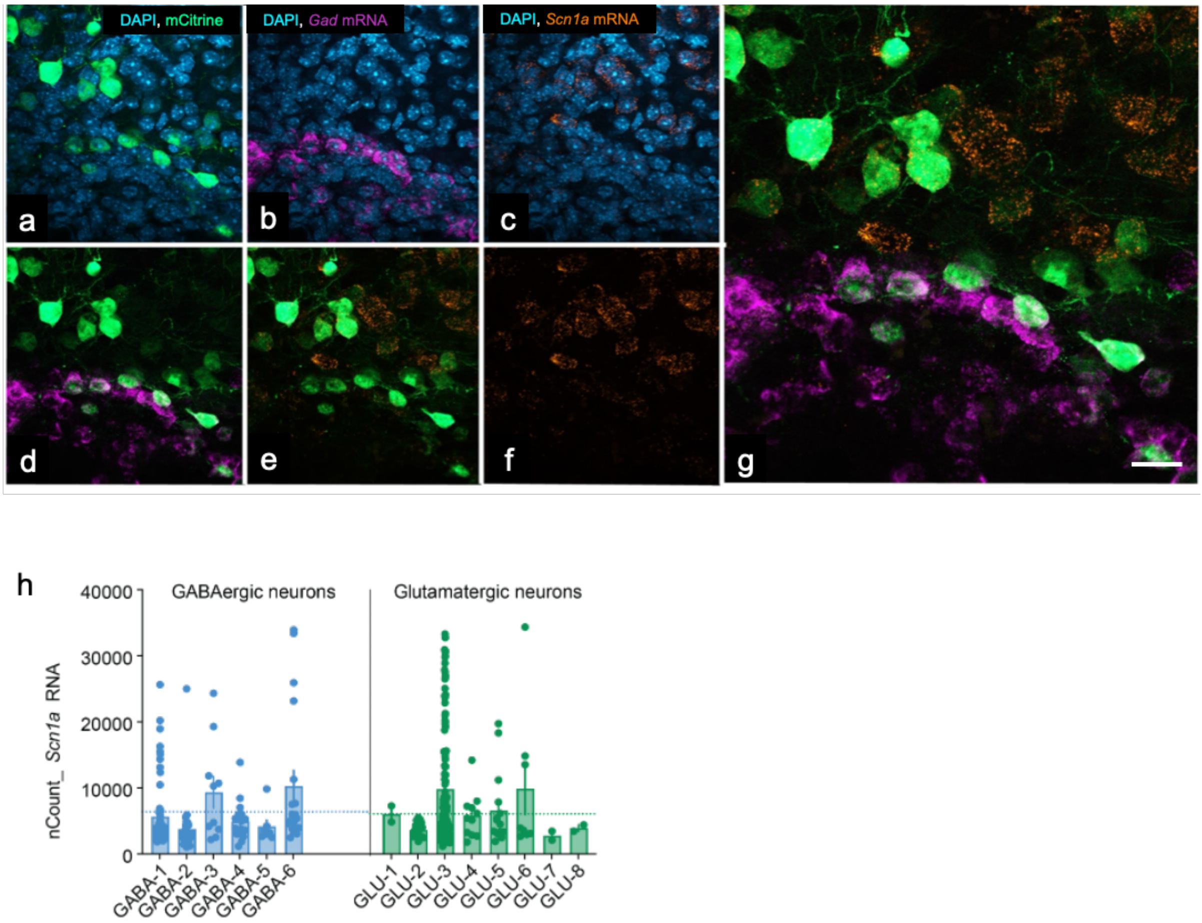

Supplementary Fig. 8

Scn1a expression in excitatory and inhibitory neurons in the hypothalamus

a-g. A Representative section of the hypothalamus of a WT mouse injected with CAV2-mCitrine. mCitrine immunofluorescence (green), and RNAScope (*Gad* in magenta, *Scn1a* in red) showing the expression of *Scn1a* mRNA in both excitatory and inhibitory neurons.

a.mCitrine and DAPI. b. *Gad* and DAPI, c. *Scn1a* and DAPI. d. mCitrine and *Gad.* e. *Scn1a* and mCitrine. f. *Scn1a.* g. mCitrine, *Gad* and *Scn1a.* Scale bar: 10 μM. Representative result from three independent experiments.

h. Single-cell RNA-sequencing data restructured based on the open-access hypothalamic single-cell RNA-seq data from Steuernagel et al. (Steuernagel et al., 2022), showing *Scn1a* transcript levels (nCount RNA) in multiple clusters of GABAergic (blue) and glutamatergic (green) neurons. The dashed lines represent the average expression level across all types of GABAergic neurons or all types of glutamatergic neurons.

Source data are provided as a Source Data file.

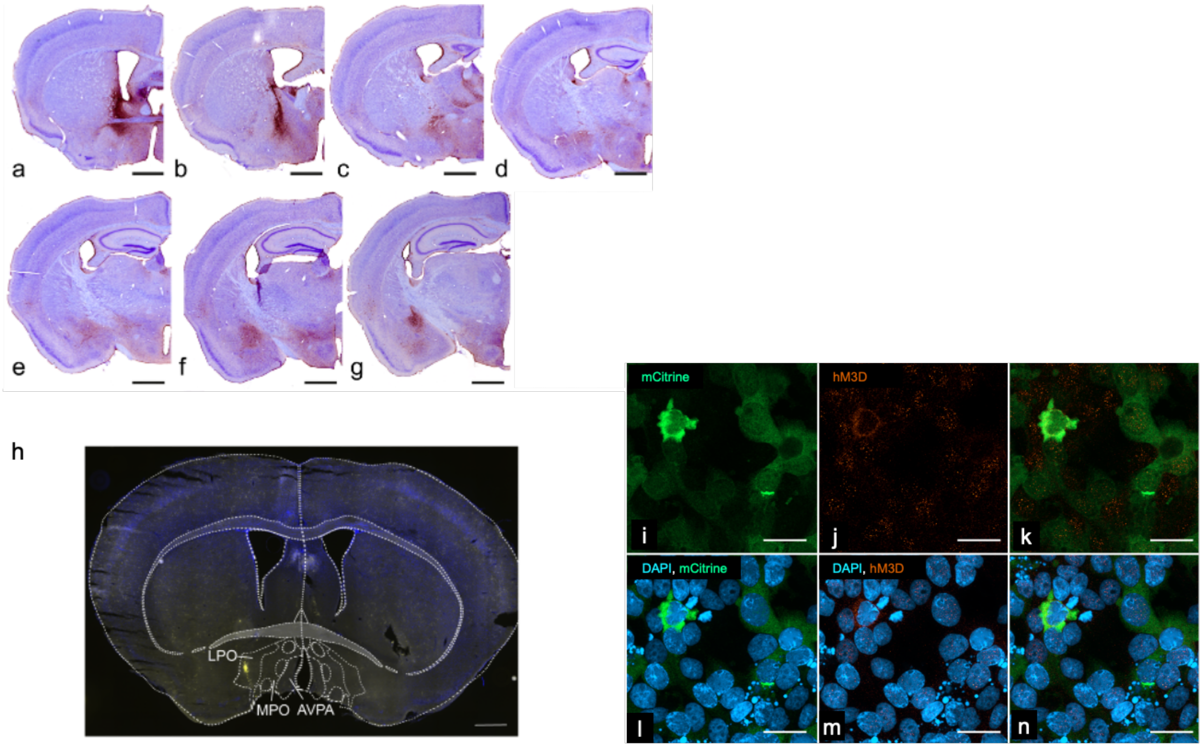

Supplementary Fig. 9

(a-g). Biodistribution of CAV2-mCitrine (in which the mCitrine reporter is driven by the. NSE promoter) following stereotaxic injection into the hypothalamic preoptic region. Due to CAV2's retrograde transport properties, injection into the hypothalamus resulted in expression in multiple regions of the hypothalamus, thalamus, amygdala, and some cortical regions. (a-g) mCitrine immunoreactivity was observed at the injection site (a), and mCitrine-positive cells and fibers were observed across the preoptic region, including the lateral and medial preoptic areas, as well as the anterior and lateral hypothalamus (a-h). Thalamic regions also contained numerous labeled cells (c-g). Some cortical labeling was observed in the agranular and piriform cortices (a-g), although the number of positive cells in those regions was relatively low. The amygdala displayed mCitrine-positive cells and fibers, mainly within the medial and basolateral nuclei. Scale bars: 1 mm. Representative result from six independent experiments.

(h). CAV2-hM3D IRES miCitrine expression in the brain. The expression of mCitrine (yellow; counterstaining with DAPI in blue) in the anterior hypothalamus. LPO: Lateral preoptic area, MPO: medial preoptic area, AVPV: anteroventral periventricular nucleus.
Scale bar: 1 mm. Representative result from six independent experiments.
(i-n). CAV2-hM3D IRES miCitrine expression in the DK-E1 cells. DK-E1 cells were seeded as a monolayer and incubated with CAV2-hM3D-IRES-mCitrine (50 particles/cell) for 24 h, followed by immunofluorescence. mCitrine immunofluorescence (green), and hM3D (red).
(i) .mCitrine; (j) hM3D; (k) mCitrine and hM4D; (l) DAPI and mCitrine; (m) DAPI and hM3D, (n) DAPI, mCitrine and hM3D). Anti-GFP (1:1000, Abcam, ab13970, RRID:AB_300798) was used to detect mCitrine. HPA024106 (1:100, Sigma) was used to detect hM3D.
Scale bar: 10 μM

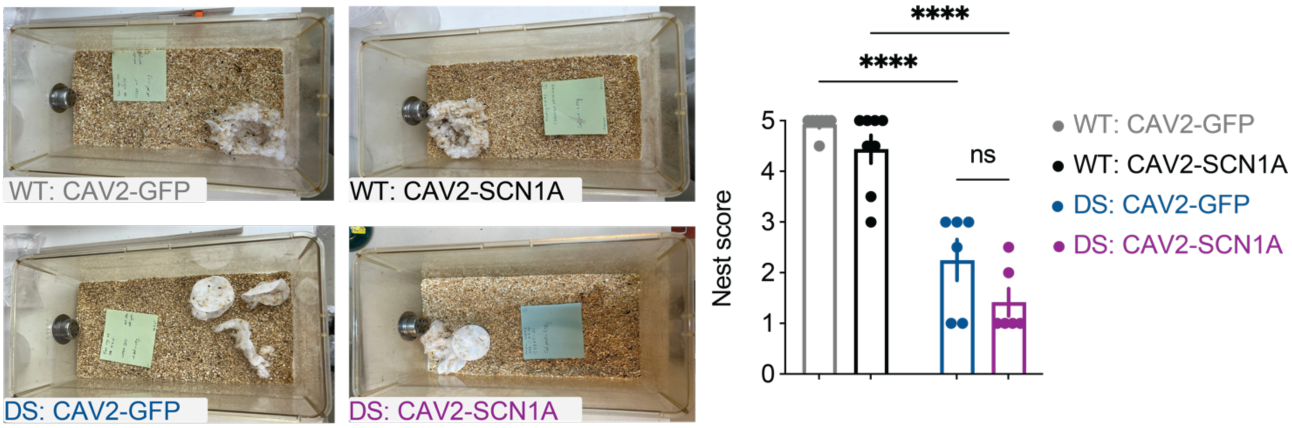

Supplementary Fig. 10

CAV2-SCN1A injection into the hypothalamus does not correct nest-building abilities in DS mice.

Examples of the nesting material after a night in the home cage (left) and nest-building scores (right) showing reduced nest-building behavior in DS mice injected with CAV2-GFP or CAV2-SCN1A (WT: CAV2-GFP, n = 7. WT: CAV2-SCN1A, n = 8. DS: CAV2-GFP, n = 6. DS: CAV2-SCN1A, n = 6. $p < 0.0001$, 2-way ANOVA. The results of Holm-Sidak post hoc analyses are depicted on the graph. Source data are provided as a Source Data file.

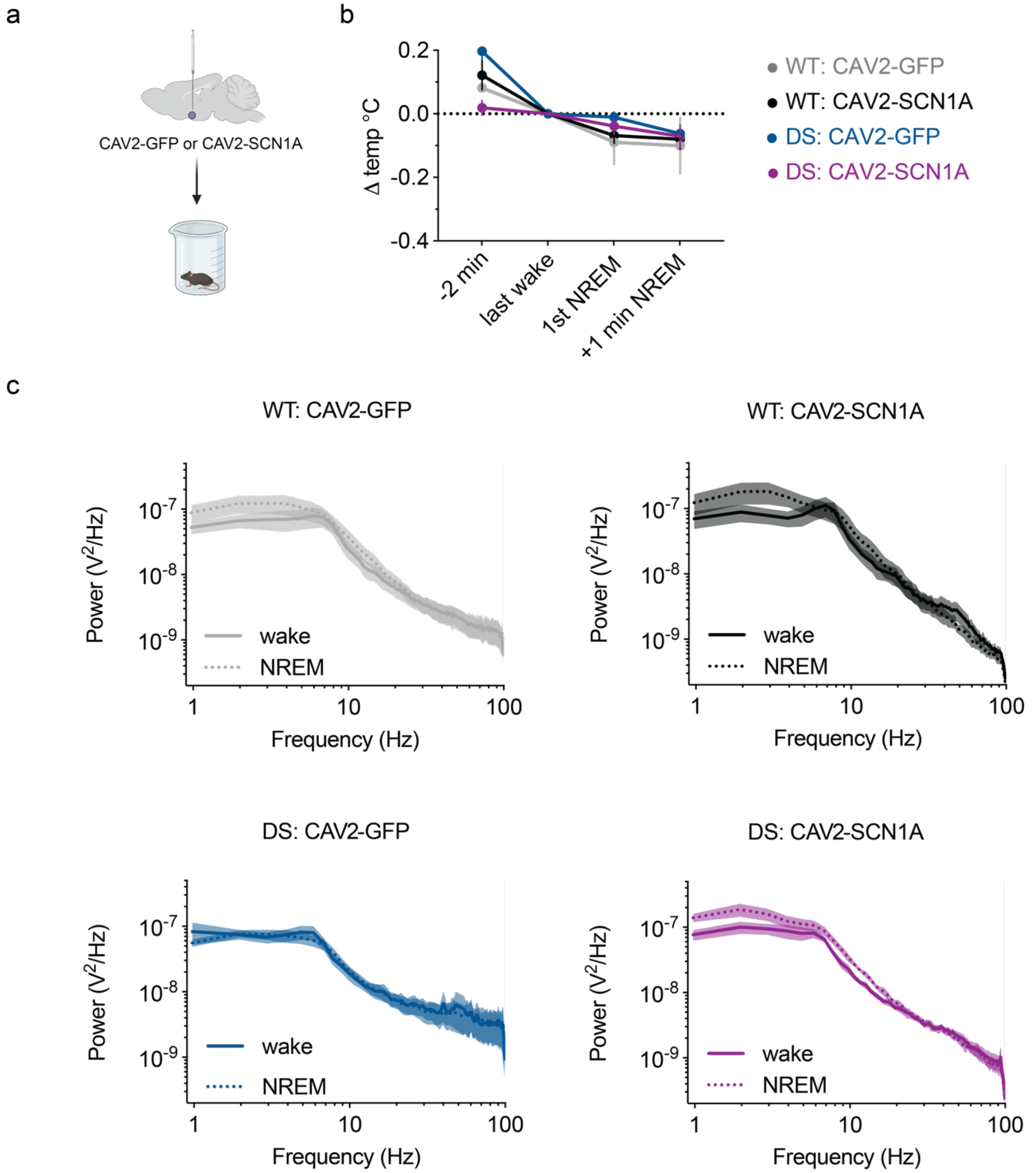

Supplementary Fig. 11
Related to Fig. 4a-e.
(a). The experimental paradigm, used for monitoring effects of hypothalamic CAV-SCN1A or control CAV-GFP expression on body core temperature changes during wake-NREM transitions, as recorded with simultaneous ECoG and temperature measurements during 3-4-h recordings at RT.
(b). The average change in core temperature at the indicated time points.
(c). ECoG PSD during wakefulness and NREM in WT: CAV2-GFP, n = 7; WT: CAV2-SCN1A, n = 6; DS: CAV2-GFP, n = 6; DS: CAV2-SCN1A, n = 5. (Two way ANOVA results for the main effect of sleep: WT: CAV2-GFP, p = 0.03; ; WT: CAV2-SCN1A, p = 0.058; DS: CAV2-GFP, p = 0.62; DS: CAV2-SCN1A, p = 0.017). Source data are provided as a Source Data file.

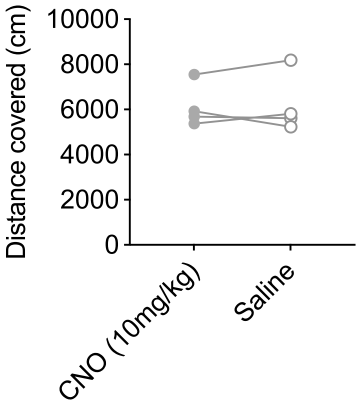

Supplementary Fig. 12
CNO administered at 10 mg/kg had no effect on the locomotor activity of WT mice (n = 4). WT mice were placed in a 50 x 50 cm open field arena 15 min after CNO injection, and their locomotor activity within the arena was recorded for 10 min. Four days later, the same mice were injected with saline, and their locomotor activity was recorded again 15 min after saline injection over 10 min. The similar distance covered under both conditions indicates that CNO at this dose does not have a non-specific sedative effect. Source data are provided as a Source Data file.
