# Supplementary material for: Disrupted temperature-sleep coupling mechanism in a Dravet syndrome mouse model
Source: Nat Commun. 2026 Feb 26;17:3232. doi: 10.1038/s41467-026-69957-1 (PMC13062047; doi:10.1038/s41467-026-69957-1)
Supplement: Supplementary file 2 — Description of Additional Supplementary Files [file 41467_2026_69957_MOESM2_ESM.pdf]

Description of Additional Supplementary Files

Supplementary Data 1 Full statistical information for all figures, including the number of mice, the statistical tests used, and exact p-values.
