# Supplementary material for: Disrupted temperature-sleep coupling mechanism in a Dravet syndrome mouse model
Source: Nat Commun. 2026 Feb 26;17:3232. doi: 10.1038/s41467-026-69957-1 (PMC13062047; doi:10.1038/s41467-026-69957-1)
Supplement: Supplementary file 4 — Reporting Summary [file 41467_2026_69957_MOESM4_ESM.pdf]

nature portfolio

Corresponding author(s): Moran Rubinstein

Last updated by author(s): 10.2.2026

Reporting Summary

Nature Portfolio wishes to improve the reproducibility of the work that we publish. This form provides structure for consistency and transparency in reporting. For further information on Nature Portfolio policies, see our Editorial Policies and the Editorial Policy Checklist.

Statistics

For all statistical analyses, confirm that the following items are present in the figure legend, table legend, main text, or Methods section.

n/a	Confirmed	
☐	☒	The exact sample size (*n*) for each experimental group/condition, given as a discrete number and unit of measurement
☐	☒	A statement on whether measurements were taken from distinct samples or whether the same sample was measured repeatedly
☐	☒	The statistical test(s) used AND whether they are one- or two-sided<br>*Only common tests should be described solely by name; describe more complex techniques in the Methods section.*
☐	☒	A description of all covariates tested
☐	☒	A description of any assumptions or corrections, such as tests of normality and adjustment for multiple comparisons
☐	☒	A full description of the statistical parameters including central tendency (e.g. means) or other basic estimates (e.g. regression coefficient) AND variation (e.g. standard deviation) or associated estimates of uncertainty (e.g. confidence intervals)
☐	☒	For null hypothesis testing, the test statistic (e.g. *F*, *t*, *r*) with confidence intervals, effect sizes, degrees of freedom and *P* value noted<br>*Give P values as exact values whenever suitable.*
☒	☐	For Bayesian analysis, information on the choice of priors and Markov chain Monte Carlo settings
☒	☐	For hierarchical and complex designs, identification of the appropriate level for tests and full reporting of outcomes
☒	☐	Estimates of effect sizes (e.g. Cohen's *d*, Pearson's *r*), indicating how they were calculated

Our web collection on statistics for biologists contains articles on many of the points above.

Software and code

Policy information about availability of computer code

Data collection	LabChart 8, Neuralynx
Data analysis	LabChart 8, Spike 2, Prism 10.4.1, Excel 16.90, MATLAB R2021b

For manuscripts utilizing custom algorithms or software that are central to the research but not yet described in published literature, software must be made available to editors and reviewers. We strongly encourage code deposition in a community repository (e.g. GitHub). See the Nature Portfolio guidelines for submitting code & software for further information.

Data

Policy information about availability of data

All manuscripts must include a data availability statement. This statement should provide the following information, where applicable:

- Accession codes, unique identifiers, or web links for publicly available datasets
- A description of any restrictions on data availability
- For clinical datasets or third party data, please ensure that the statement adheres to our policy

The source file contains all data presented in the figures, and references to this file have been added to all relevant figures

Research involving human participants, their data, or biological material

Policy information about studies with human participants or human data. See also policy information about sex, gender (identity/presentation), and sexual orientation and race, ethnicity and racism.

Reporting on sex and gender	NA
Reporting on race, ethnicity, or other socially relevant groupings	NA
Population characteristics	NA
Recruitment	NA
Ethics oversight	NA

Note that full information on the approval of the study protocol must also be provided in the manuscript.

Field-specific reporting

Please select the one below that is the best fit for your research. If you are not sure, read the appropriate sections before making your selection.

- [x] Life sciences
- [ ] Behavioural & social sciences
- [ ] Ecological, evolutionary & environmental sciences

For a reference copy of the document with all sections, see nature.com/documents/nr-reporting-summary-flat.pdf

Life sciences study design

All studies must disclose on these points even when the disclosure is negative.

Sample size	No statistical methods were used to predetermine sample sizes. Sample sizes for all experiments were chosen based on common practice and established standards in the field, as well as on prior experience with similar experimental paradigms and effect sizes. The selected sample sizes are comparable to those used in previously published studies employing similar methodologies and were sufficient to detect biologically meaningful differences between groups.
Data exclusions	Mice in which the ECoG or LFP recordings electrodes were bad, and the signal was noisy despite the filter were excluded from the analyses
Replication	The number of examined mice is listed on the Fig. legends and in Supplementary Data 1
Randomization	We used the whole litter, including WT and DS littermate for the exp.
Blinding	Blinding was not feasible because, even without reference to tag numbers, DS mice could be readily distinguished from their WT littermates based on their characteristic motor hyperactivity. Data analysis was likewise not blinded, as DS mice exhibited prominent interictal spikes that were readily apparent in the ECoG or LFP recordings. Mice were tagged, weaned, and genotyped at postnatal day 21 (P21), and entire litters were used for the experiments.

Reporting for specific materials, systems and methods

We require information from authors about some types of materials, experimental systems and methods used in many studies. Here, indicate whether each material, system or method listed is relevant to your study. If you are not sure if a list item applies to your research, read the appropriate section before selecting a response.

Materials & experimental systems

n/a	Involved in the study	
[ ]	[x]	Antibodies
[x]	[ ]	Eukaryotic cell lines
[x]	[ ]	Palaeontology and archaeology
[ ]	[x]	Animals and other organisms
[ ]	[ ]	Clinical data
[x]	[ ]	Dual use research of concern
[x]	[ ]	Plants

Methods

n/a	Involved in the study	
[x]	[ ]	ChIP-seq
[x]	[ ]	Flow cytometry
[x]	[ ]	MRI-based neuroimaging

Antibodies

Antibodies used: chicken anti-GFP (1:1000, Abcam, ab13970, RRID:AB_300798), Alexa Fluor® 488 donkey anti-chicken IgG (1:200, Jackson ImmunoResearch, Cat#703-545-155, RRID:AB_2340375)

Validation: The chicken anti-GFP antibody (Abcam, ab13970, RRID:AB_300798) was validated by the manufacturer for immunofluorescence and has been extensively used in the literature for specific detection of GFP or mCitrine. In our experiments, the antibody produced specific labeling, with no detectable signal observed in naïve animals.

Animals and other research organisms

Policy information about studies involving animals; ARRIVE guidelines recommended for reporting animal research, and Sex and Gender in Research

Laboratory animals: WT and DS mice harboring the global Scn1aA1783V/WT mutation were generated by crossing conditional floxed Scn1aA1783V/WT males (The Jackson Laboratory; stock #026133, C57BL/6J) with CMV-Cre females (The Jackson Laboratory; stock #006054, C57BL/6J), as described before (Fadila et al., 2020, 2023; Almog et al., 2021). The mice were kept on the pure C57BL/6J genetic background. Mice were housed in a standard animal facility at a constant temperature of 22°C, on a 12-hour light/dark cycle, with ad libitum access to food and water.
WT and DS mice at the age of P21-P25 were used for the data in Fig. 1
WT and DS mice during their 4th week (P21-P28) were used in Fig. 2A-F.
5-7-week-old WT and DS mice were used for the rest of the Figs (Fig 2g-l, Figs. 3-5).

Wild animals: The study did not involve wild animal

Reporting on sex: Both male and female mice were tested, and the data were pooled together. There are no reports of variation in brain activity between DS males and females . As we used the whole litter for experiments, there was no specific selection for either sex

Field-collected samples: The study did not involve data collected from the field

Ethics oversight: All animal experiments were approved by the Institutional Care and Use Committee of Tel Aviv University
protocols TAU - MD - IL - 2305 - 130 - 4, TAU - MD - IL - 2601 - 104 - 4

Note that full information on the approval of the study protocol must also be provided in the manuscript.

Clinical data

Policy information about clinical studies

All manuscripts should comply with the ICMJE guidelines for publication of clinical research and a completed CONSORT checklist must be included with all submissions.

Clinical trial registration: NA

Study protocol: NA

Data collection: NA

Outcomes: NA

Plants

Seed stocks: NA

Novel plant genotypes: NA

Authentication: NA
